# Reply to: ‘Reconstructed evolutionary patterns from crocodile-line archosaurs demonstrate the impact of failure to log-transform body size data’

**DOI:** 10.1038/s42003-022-03072-x

**Published:** 2022-02-25

**Authors:** Maximilian T. Stockdale, Michael J. Benton

**Affiliations:** 1 School of Geographical Sciences, University of Bristol, Bristol, BS8 1RL UK; 2 School of Earth Sciences, University of Bristol, Bristol, BS8 1RL UK

**Keywords:** Palaeontology, Biodiversity

**replying to** R. Benson et al. *Communications Biology* 10.1038/s42003-022-03071-y (2022)

In our recent analysis of body size evolution in the Pseudosuchia^1^, we concluded a variable rate model significantly outperforms a random walk, and that evolutionary rates show interactions between body size evolution and the environment. Benson et al.^2^ express concern that these findings are inconsistent with previous work^3,4^, which have found an Ornstein-Uhlenbeck (OU) model to be well supported. They attribute this to our not having used a log transformation, and propose that this undermines our findings due to the effects of relative scaling. Benson et al. raise some important points; however, there is insufficient evidence to accept the revised conclusions that they propose. In this revision of our analysis, we conclude that there are too few exceptionally large taxa to change the outcomes of our analysis, and that the strength of any Ornstein–Uhlenbeck process is negligible. Simulations replicate the findings of Benson et al using random data, suggesting log transformation may inflate and suppress some model likelihoods.

Our analysis has a number of features that distinguish it from previous publications. Our phylogenetic tree incorporates molecular data, causing substantive changes to the topology of the crocodile crown-group^5^. Models have also been fitted using a Bayesian model-fitting algorithm. This makes comparisons with previous work difficult. Previous publications have not been consistent in recovering support for the OU model, depending on what body size proxy has been used^3^. Therefore the claim by Benson et al. that our analysis is not consistent with previous publications is difficult to justify.

We concede that the high evolutionary rates observed in the largest taxa are likely to be a result of scaling bias, and this is a more likely explanation than was speculated in our manuscript. However, the range is not a meaningful measure of variance in a normal distribution, and common ancestors must also be included. In practice, the fraction of very large taxa is small. To demonstrate this we examined the distributions of two well-represented characters from our original dataset (Supplementary Data 1), skull width and the length from the posterior-most point of the supraoccipital to the anterior-most point of the frontal. We added estimated common ancestors using an ancestral state reconstruction, using the APE library for R^6^. The distributions of these characters reveal that 80% of skull width measures, and 83% of frontal-supraoccipital length measures, were within 10cm of the median. Therefore, the overall variance within the body size data is modest.

Figure 1a of Benson et al. shows a divergence of transformed and untransformed metrics from a linear relationship. However, much of this divergence is driven by a minority of extremely large taxa (Supplementary Information Document 1). Exceptionally large taxa are too few in number to significantly change the outcomes of our analysis. This can be demonstrated by replicating our analysis with the exceptionally large and small taxa removed. We created a new dataset with the largest 20% of taxa and the smallest 10% of taxa removed. Random walk and variable rates models were then fitted to this revised dataset using BayesTraits version 3^7^. These models were then compared using Bayes factors. The variable rate model yielded a Bayes factor of 41 when compared to Brownian motion, indicating strong support for the variable rate model. Therefore support for the variable rate model cannot be attributed to scaling bias in exceptionally large taxa. Plotting branch rates on a phylogenetic tree (Fig. 1) reveals a rate pattern similar to our original analysis^1^, and our original observations still apply. In particular, evolutionary rate shifts do not appear to be associated with phylogenetic groups. Instead, there is a background of low evolutionary rates, punctuated by discrete increases in evolutionary rate. This fully supports our original conclusion that body size evolution follows a pattern of punctuated equilibrium.

**Fig. 1:**
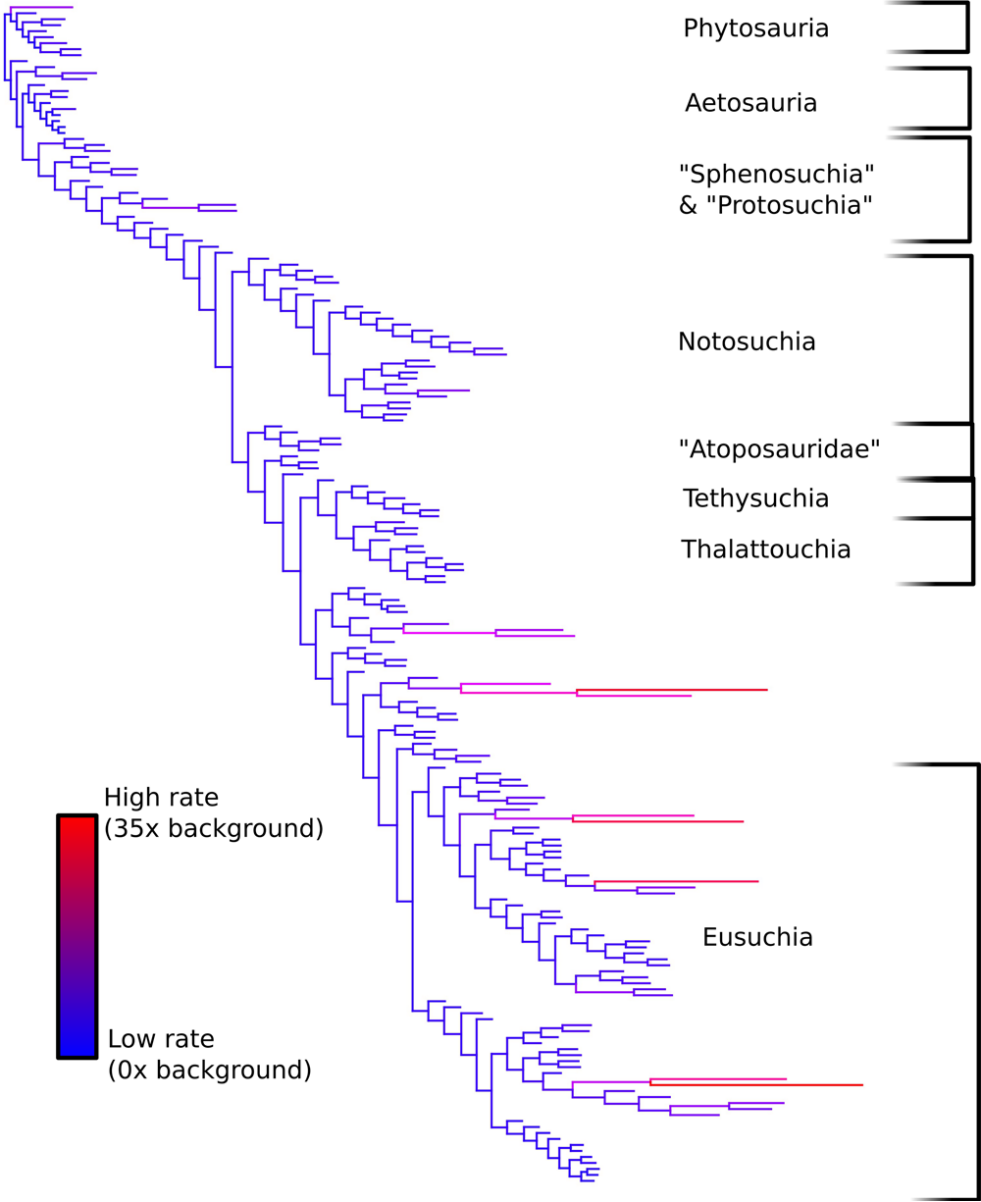
Variable evolutionary rates plotted on a phylogenetic tree, derived from a dataset removed of exceptionally large and small taxa. Branches displaying a higher evolutionary rate are longer and shown in shades of purple and red. Branches displaying a lower evolutionary rate are shorter and shown in shades of blue. This plot compares favourably with Fig. 1 of our original analysis. The tree is dominated by low evolutionary rates that are interrupted by episodic increases. This supports our original conclusion that crocodile body size evolution follows a punctuated equilibrium model.

The revised body size dataset was used to plot a time series of body size variance (Fig. 2), comparable with Fig. 3 from our original manuscript^1^. This revised time series shows striking similarities with the original across all three approaches to curve estimation. Our manuscript describes periods where body size variance remains steady, punctuated by steps up and down in variance during the Late Triassic, Middle Jurassic, Palaeogene and Neogene. These features of our original curve are clearly visible in the revised curve shown in Fig. 2. Therefore the distribution of body size variance through time observed in our analysis is not an artefact caused by including exceptionally large taxa. The distribution of variance through time shown in Fig. 2c shows periods of relative stability, interrupted occasionally by sharp increases and decreases in body size disparity. This strongly supports the pattern of punctuated equilibrium proposed in our original manuscript^1^.

**Fig. 2:**
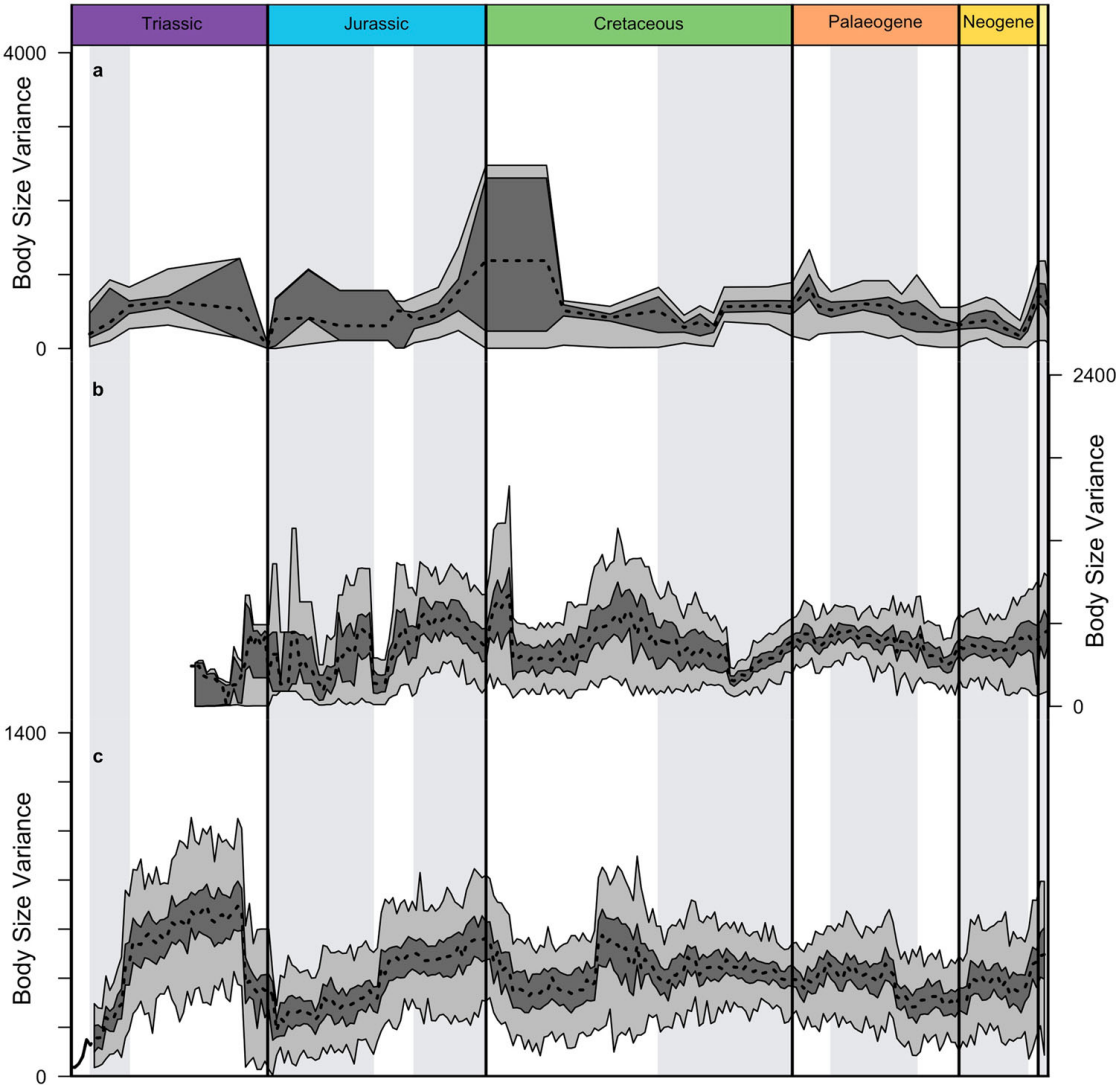
Body size variance through time estimated using a dataset removed of exceptionally large taxa. Three curve estimation approaches are shown: **a** actual taxa binned at the resolution of stratigraphic stages; **b** actual taxa plus ghost ranges inferred using phylogenetic tree branch lengths; **c** actual taxa plus ghost ranges plus phylogenetically reconstructed body size values of inferred common ancestors. Data has been bootstrapped in a similar fashion to Fig. 3 of our original manuscript. Upper and lower quartiles of bootstrap values are shown in dark grey. The range of bootstrap values is shown in light grey. These curves compare favourably with our original publication and retain their most important features.

**Fig. 3:**
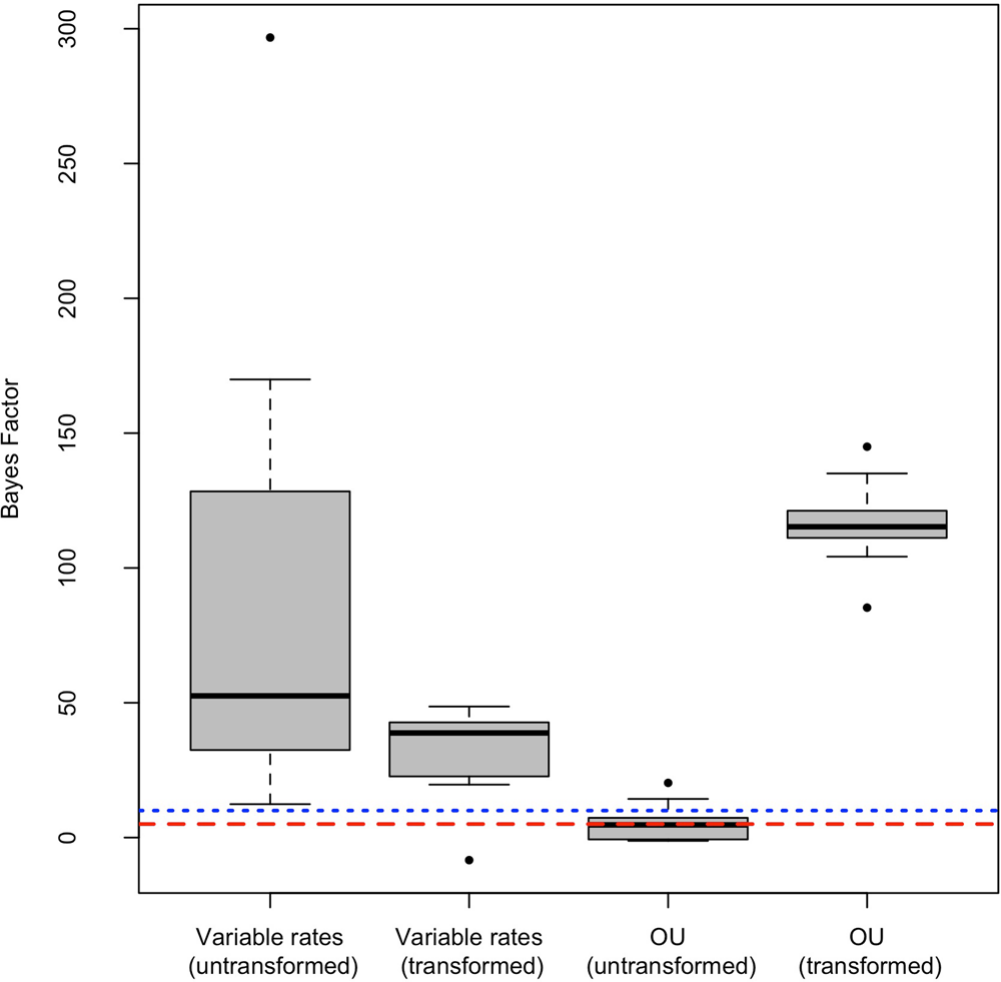
Demonstrating the effects of log transformations on support for the variable rates and OU models using random data. Centre lines of each series indicate the median; boxes in grey show upper and lower quartiles. Whiskers indicate interquartile ranges, with points indicating outliers. Moderate support, indicated by a Bayes factor of 5 or more, is indicated by the red dashed line. Strong support, indicated by a Bayes factor of 10 or more, is indicated by the blue dotted line. This demonstrates that log transformation can inherently suppress support for variable rate models, and promote support for OU models.

The comments by Benson et al. make no reference to published concerns about the OU model, in particular its propensity to give false positive results^2^. A previous analysis^8^ has recommended that OU models should not be fitted to datasets with fewer than 200 taxa without a Bayesian model-fitting approach. Not all previous publications meet these criteria^3^. Even so, false-positive rates of 100% have been reported in trees with as many as 1000 taxa when there are high rates of measurement error^8^, even with a Bayesian model-fitting approach. Body size measurement error in fossil datasets can be assumed to be high due to the effects of digenesis and other factors such as imputation error, morphological variation, ontogeny and sexual dimorphism. The OU model includes a parameter to indicate the strength of selection towards an optimum trait value, known as alpha. The *α*-value can range from 0 to infinity^8^. We replicated the analysis as described by Benson et al., using log-transformed measurements and fitted an OU model using Bayestraits. The mean *α*-value across all iterations of the MCMC chain came to 0.016. It has been advised^8^ that the *α*-value should be expressed relative to the total height of the tree and expressed as the phylogenetic half-life^9^. An *α*-value of 0.016 is equal to a half-life of 43.3 million years. This is relatively short compared to the phylogenetic tree, superficially suggesting a realistic OU process. However, this is considerably longer than the average branch length, around 10 million years, and longer than 89% of all branches on the tree. Notwithstanding the risk of false-positive results, the contribution of an OU process is clearly extremely slow. Further, this half-life is as long, or considerably longer than major subclades within the Pseudosuchia, such as the Notosuchia and Thalattosuchia. These groups have highly distinctive ecomorphologies, and it is unrealistic to assume that OU parameters would be continuous between them.

Log-transformation changes the distribution and variance of data. Benson et al make an assumption that this transformation does not inherently promote or suppress support for particular evolutionary regimes. The effects of log transformations on model likelihoods can be explored using simulations. We simulated ten random phylogenetic trees with 200 tips each, and ten sets of random trait data with an arbitrary mean trait value of 200, and standard deviations ranging from 5 to 50 (Supplementary Data 2). These random trait data represent a hypothetical continuous character. They do not represent body size specifically, and these simulations do not make inferences about body size evolution. Log transformations were then applied to duplicates of these trait datasets. We fitted random walk, variable rates and OU models to each tree using BayesTraits, once using the untransformed trait data, and repeated using logged trait data. The performance of the variable rates and OU models relative to the random walk model were compared using Bayes factors (Fig. 3). The Bayes factor of the variable rate model using logged data is lower than that of the untransformed data. This suggests that log transformation can suppress support for the variable rate model. The Bayes factors of OU models fitted using untransformed data show only modest support relative to a random walk. By contrast, the Bayes factors of models fitted using logged data show very strong support for the OU model over a random walk. This is despite the data being random and not generated using an Ornstein–Uhlenbeck process. Therefore the lack of support for the variable rate model and increased support for the OU model described by Benson et al. seems likely to be a direct result of the log transformation. These simulations do not question the efficacy of log transformation in correcting scaling bias. Nor do they suggest that scaling should not be accounted for when fitting phylogenetic models. However, they do suggest that OU models in particular should be only be accepted with caution when using log-transformed traits.

The remarks by Benson et al. have highlighted the importance of scaling in the analysis of body size evolution. We concede that scaling was not sufficiently discussed in our analysis, and that this explains the disproportionately high rates observed in very large taxa. We also concede that our rationale for not log transforming the data was incomplete. However, there are too few very large taxa to significantly change our conclusions, and there is not sufficient evidence to accept the alternative conclusions proposed.

## Reporting summary

Further information on research design is available in the Nature Research Reporting Summary linked to this article.

## Supplementary information

Supplementary Information

Description of Additional Supplementary Files

Supplementary Data 1

Supplementary Data 2

Reporting Summary

## Data Availability

All the data used in this analysis are included in the supplementary data files included with this publication.
