# Supplementary material for: Reply to: ‘Reconstructed evolutionary patterns from crocodile-line archosaurs demonstrate the impact of failure to log-transform body size data’
Source: Commun Biol. 2022 Feb 25;5:170. doi: 10.1038/s42003-022-03072-x (PMC8881626; doi:10.1038/s42003-022-03072-x)
Supplement: Supplementary file 1 — Supplementary Information [file 42003_2022_3072_MOESM1_ESM.pdf]

Supplementary Information 1: Systematic comparison of transformed and untransformed body size metrics

The divergence of transformed and untransformed body size metrics is a core argument in the comments of Benson et al. This argument relies heavily on figure 1a of their manuscript, which compares the untransformed body size metric used by Stockdale and Benton (2021) with log-transformed skull length. While we recognise the theoretical premise of this argument, this comparison is misleading and exaggerates the divergence of transformed and untransformed variables in this case.

The curved relationship between transformed and untransformed variables in figure 1a of Benson et al. is being driven by a minority of apparently large taxa. When these extremely large taxa are removed from the data set, the relationship between transformed and untransformed variables is near-linear (Supplementary Fig. 1). To demonstrate this, we implemented a linear regression using the PC1 size index using transformed variables, and the PC1 size index using untransformed variables. This recovered an R-squared value of 0.57, and a p-value of $p=6.4x10^{-38}$. This indicates a statistically significant linear relationship. The residual errors of this model were subjected to a Shapiro-Wilk test for normalcy. This recovered a test statistic of 0.9, and returned a p-value of $p=4.1x10^{-10}$, indicating a very high degree of residual normalcy. The linear regression model was analysed further using a Breush-Pagan test for heteroscedasticity, which returned a p-value of 0.13. This confirms that there is not statistically significant heteroscedasticity in this model, fulfilling the requirements of linear regression. Therefore within the size range of this revised dataset, the omission of a log transformation does not introduce statistically significant systematic error.

The linear regression analysis shows that the PC1 size index with untransformed variables performs well compared to other body size metrics. For example, we implemented linear regression using the variables shown in Benson et al. Figure 1b, PCA scores from transformed variables against log skull length. The Breusch-Pagan test recovered statistically significant heteroscedasticity, with a p-value of p=0.014. This suggests that skull length introduces a statistically significant systematic error, even when log transformed. This is to be expected, since the aspect ratio of crocodile skulls varies widely depending on the relative length of the snout. Using skull length as a body size indicator can be expected to over-estimate body size in long-snouted taxa, and under-estimate body size in short-snouted taxa.

The dependence of skull length upon snout elongation can be quantified by comparing the distribution of skull length with skull aspect ratio. We calculated the aspect ratio of all the taxa in our dataset (Supplementary Data 1) by dividing the skull length of all taxa by their corresponding skull width. This was then plotted against log skull length (Supplementary Fig. 2a). A linear regression of these data confirmed a statistically significant linear relationship, with a p-value of $p=1.19x10^{-15}$. The slope coefficient for this regression was found to be 0.43, indicating a 43% increase in in log skull length for every unit increase in skull aspect ratio. The median aspect ratio of the complete dataset was found to be 2.01, while approximately 14% of taxa in the data set have an aspect ratio of 3.02 or higher, indicating a 50% inflation of apparent body size in

these taxa. Further, around 4% of taxa in the dataset have an aspect ratio of 4.02 or higher. This suggests that log skull length can double the apparent body size if the snout is highly elongated. We performed a similar comparison using log skull width (Supplementary Fig. 2b). This analysis yielded no significant relationship, and the regression line is almost flat, with a slope coefficient of 0.02. Therefore log skull width is independent of snout elongation.

The dependence of log skull length on snout elongation raises further issues with figure 1a of Benson et al. As stated previously, the curved relationship between transformed and untransformed body size metrics is driven by a minority of apparently large taxa. However, the dependence of skull length on skull aspect ratio suggests that the body sizes in this minority have been inflated, exaggerating the apparent divergence between transformed and untransformed body size metrics. This can be demonstrated by comparing the performance of nth power curves relative to simple linear models (Supplementary Fig. 3). We fitted an nth power curve to the variables shown in figure 1a of Benson et al. This returned a fitted curve where $y = x^{3.4}$- 73.3, and a log-likelihood of -719.07. These variables were then fitted with a simple linear model, returning a fitted line where y=x56.6-192.1, and a log-likelihood of -807.7. These model fitting steps were then repeated, substituting log skill length for log skull width. This returned a fitted nth-power curve where y=$x^{3.8}$-55.7, and a fitted line where y=x63.4 – 168.52. These fitted models returned log-likelihoods of -781.43 and -831.41 respectively. For each body size proxy we compared the corresponding pair of models using Bayes factors. The Bayes factor for models using log skull length was 177.26, while the Bayes factor for models using log skull width was 99. Therefore the nth power curve outperforms the straight line model to a much greater degree for log skull length than it does log skill width.

Both Bayes factors strongly favour the nth-power curve over linear models, which supports the arguments made by Benson et al. However, the difference in Bayes factors reveals that the relationship between transformed and untransformed body size metrics may be more or less linear depending on what proxy is being used. The body size metric used by Benson et al. introduces systematic error, which exaggerates the divergence of transformed and untransformed data. When a more suitable body size metric is used, this divergence is lessened considerably. This observation does not undermine the points made by Benson et al. However, they do suggest that figure 1a of Benson et al. is misleading and misrepresents the limitations of untransformed data in this case.

The analysis in this reply features a revised dataset removed of the smallest 10% and the largest 20% of taxa (see main text). The efficacy of this approach can be demonstrated using the methodology of Benson et al., but substituting log skull length for a proxy that is independent of snout elongation. Skull width data was removed of the largest 10% and the smallest 20% of taxa. These data were log transformed and plotted against the PCA size index using untransformed variables (Supplementary Fig. 4). These points were fitted with a linear model and an *n*th-power curve. The linear model returned a log-likelihood of -506.8, and the *n*th-power curve a log likelihood of -507.6. These likelihoods were compared using Bayes factors, returning a value of 1.58. This suggests that the difference in performance between these two models is negligible.

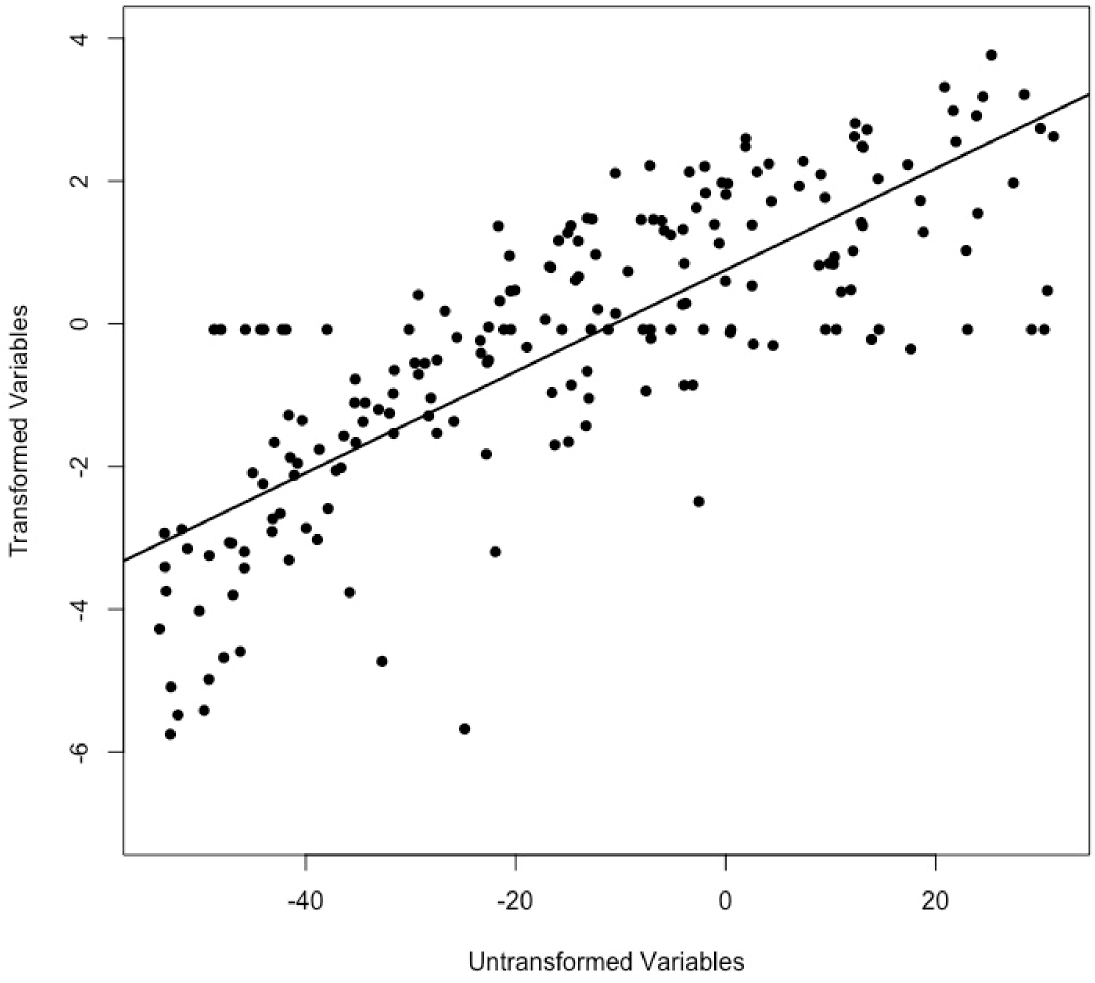

Supplementary Figure 1: Regression of PC1 size indices using transformed and untransformed variables, and a revised dataset that excludes exceptionally large and small taxa. The linear regression analysis was found to be statistically significant, with normally distributed residual errors and without statistically significant heteroscedasticity.

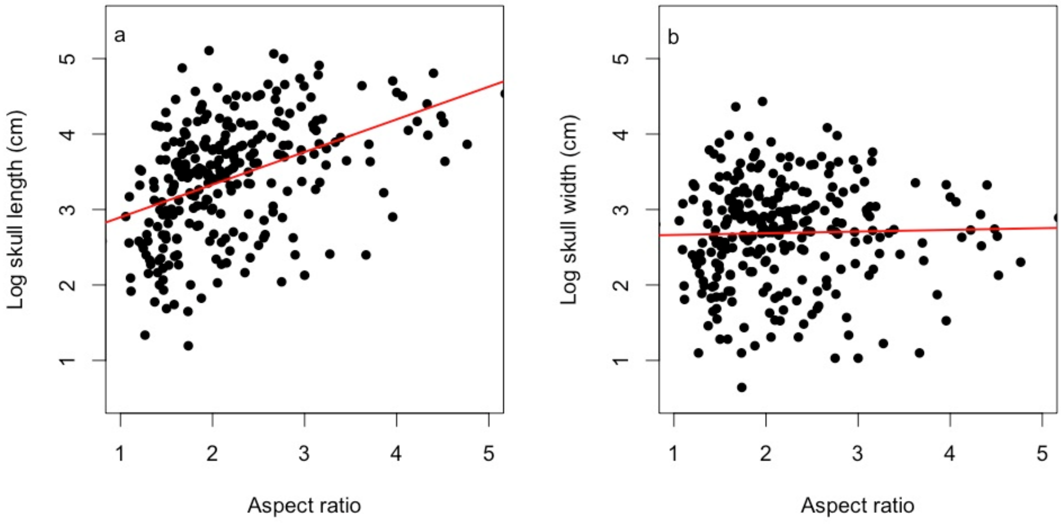

Supplementary Figure 2: Comparison of log body size metrics with skull aspect ratio. (**a**) The body size metric used in Fig. 1a of Benson et al, log skull length, shows a statistically significant linear relationship with aspect ratio. This shows that log skull length will systematically overestimate body size as aspect ratio increases. (**b**) shows similar comparison with log skull width, which shows no such relationship.

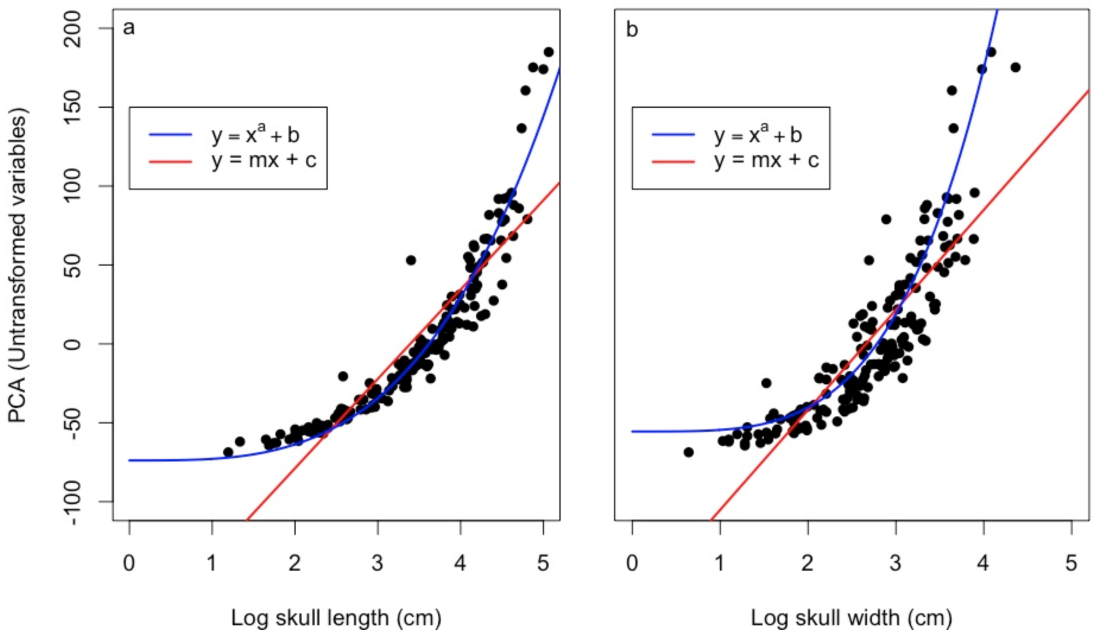

Supplementary Figure 3: Comparing the relative performance of *n*th power curve and straight line models in predicting the divergence of transformed and untransformed body size metrics. The performance of the *n*th power curve relative to the straight line in (**a**) is much higher than models shown in (**b**), suggesting that log skull length exaggerates the divergence of transformed and untransformed body size metrics from a linear relationship.

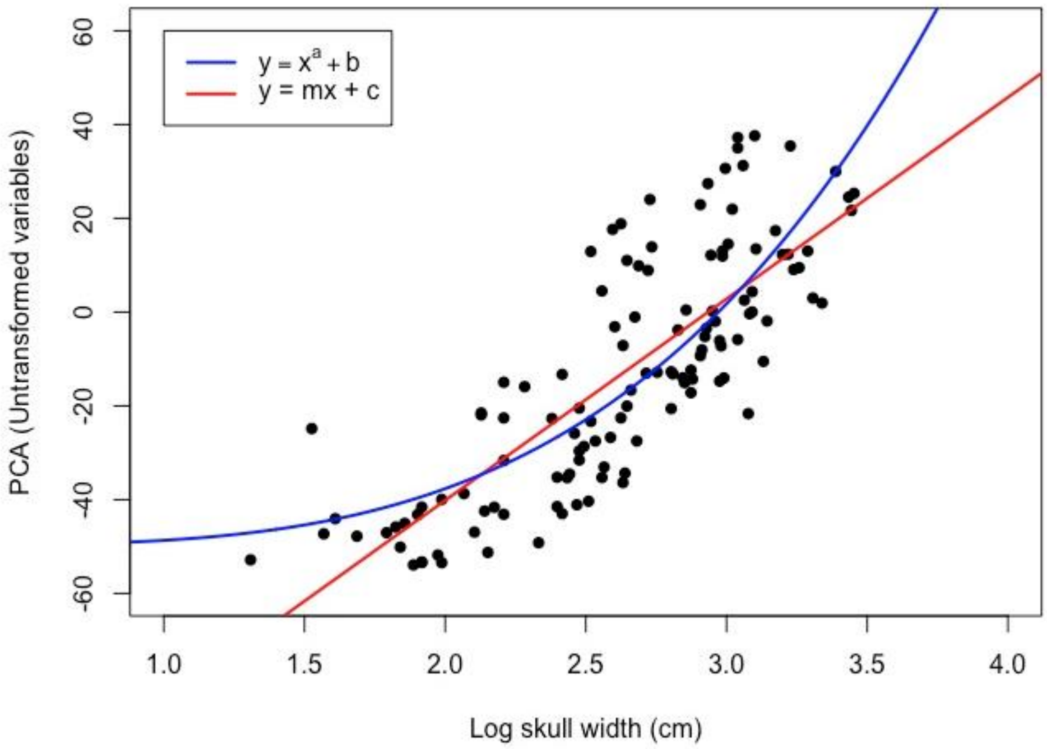

Supplementary Figure 4: Log skull width plotted against the PC1 body size index using untransformed variables. These data exclude exceptionally large and small taxa, which may drive the divergence of transformed and untransformed body size metrics. The transformed body size metric has been chosen for its independence from snout elongation. Bayes factors suggest that the *n*th power curve, shown in blue, does not fit these data significantly better than the linear regression, shown in red.
