# Supplementary material for: Reply to: ‘Reconstructed evolutionary patterns from crocodile-line archosaurs demonstrate the impact of failure to log-transform body size data’
Source: Commun Biol. 2022 Feb 25;5:170. doi: 10.1038/s42003-022-03072-x (PMC8881626; doi:10.1038/s42003-022-03072-x)
Supplement: Supplementary file 2 — Description of Additional Supplementary Files [file 42003_2022_3072_MOESM2_ESM.pdf]

Description of Additional Supplementary Files

File name: Supplementary Data 1

Description: This data file contains trait data following the imputation procedure described in Stockdale & Benton (2021).

File name: Supplementary Data 2

Description: This file contains files relating to the simulations described in the manuscript. This includes simulated trees, simulated trait data, BayesTraits log files.
