# Supplementary material for: Reply to: ‘Reconstructed evolutionary patterns from crocodile-line archosaurs demonstrate the impact of failure to log-transform body size data’
Source: Commun Biol. 2022 Feb 25;5:170. doi: 10.1038/s42003-022-03072-x (PMC8881626; doi:10.1038/s42003-022-03072-x)
Supplement: Supplementary file 5 — Reporting Summary [file 42003_2022_3072_MOESM5_ESM.pdf]

nature research

Corresponding author(s): Maximilian T. Stockdale

Last updated by author(s): Oct 21, 2021

Reporting Summary

Nature Research wishes to improve the reproducibility of the work that we publish. This form provides structure for consistency and transparency in reporting. For further information on Nature Research policies, see our Editorial Policies and the Editorial Policy Checklist.

Statistics

For all statistical analyses, confirm that the following items are present in the figure legend, table legend, main text, or Methods section.

n/a	Confirmed	
x		The exact sample size (n) for each experimental group/condition, given as a discrete number and unit of measurement
x		A statement on whether measurements were taken from distinct samples or whether the same sample was measured repeatedly
x		The statistical test(s) used AND whether they are one- or two-sided<br>*Only common tests should be described solely by name; describe more complex techniques in the Methods section.*
x		A description of all covariates tested
	x	A description of any assumptions or corrections, such as tests of normality and adjustment for multiple comparisons
	x	A full description of the statistical parameters including central tendency (e.g. means) or other basic estimates (e.g. regression coefficient) AND variation (e.g. standard deviation) or associated estimates of uncertainty (e.g. confidence intervals)
	x	For null hypothesis testing, the test statistic (e.g. F, t, r) with confidence intervals, effect sizes, degrees of freedom and P value noted<br>*Give P values as exact values whenever suitable.*
	x	For Bayesian analysis, information on the choice of priors and Markov chain Monte Carlo settings
x		For hierarchical and complex designs, identification of the appropriate level for tests and full reporting of outcomes
x		Estimates of effect sizes (e.g. Cohen's d, Pearson's r), indicating how they were calculated

Our web collection on statistics for biologists contains articles on many of the points above.

Software and code

Policy information about availability of computer code

Data collection	Some data used in this analysis was published in our previous publication, Stockdale & Benton 2021 (Nature Communications Biology). The remaining data was simulated specifically for this analysis and is included in the the simulation.
Data analysis	Data was analyzed using R package APE and the free software application BayesTraits, which are cited in the manuscript

For manuscripts utilizing custom algorithms or software that are central to the research but not yet described in published literature, software must be made available to editors and reviewers. We strongly encourage code deposition in a community repository (e.g. GitHub). See the Nature Research guidelines for submitting code & software for further information.

Data

Policy information about availability of data

All manuscripts must include a data availability statement. This statement should provide the following information, where applicable:

- Accession codes, unique identifiers, or web links for publicly available datasets
- A list of figures that have associated raw data
- A description of any restrictions on data availability

All the data used in this analysis are included in the supplementary data files included with this publication.

Field-specific reporting

Ecological, evolutionary & environmental sciences study design

All studies must disclose on these points even when the disclosure is negative.

Study description	Reanalysis of published data using revised numerical methodology.
Research sample	Analysis uses secondary data published in a previous publication.
Sampling strategy	Analysis uses secondary data published in a previous publication.
Data collection	Analysis uses secondary data published in a previous publication.
Timing and spatial scale	Not applicable
Data exclusions	A core argument of our manuscript is that our findings were not driven by exceptionally large taxa. To demonstrate that very large and small bodied taxa are excluded from our analysis as described in our manuscript.
Reproducibility	Methods are described in detail. All software used is cited in the manuscript and freely available to download. All data used in the analysis is provided in the supplementary information.
Randomization	Not applicable
Blinding	Not applicable

Did the study involve field work? ☐ Yes ☒ No

Reporting for specific materials, systems and methods

We require information from authors about some types of materials, experimental systems and methods used in many studies. Here, indicate whether each material, system or method listed is relevant to your study. If you are not sure if a list item applies to your research, read the appropriate section before selecting a response.

Materials & experimental systems

n/a	Involved in the study
☒	☐ Antibodies
☒	☐ Eukaryotic cell lines
☐	☒ Palaeontology and archaeology
☒	☐ Animals and other organisms
☒	☐ Human research participants
☒	☐ Clinical data
☒	☐ Dual use research of concern

Methods

n/a	Involved in the study
☒	☐ ChIP-seq
☒	☐ Flow cytometry
☒	☐ MRI-based neuroimaging

Palaeontology and Archaeology

Specimen provenance	Data used in the analysis is published in a previous publication.
Specimen deposition	Analysis uses secondary data published in a previous publication.
Dating methods	*If new dates are provided, describe how they were obtained (e.g. collection, storage, sample pretreatment and measurement), where they were obtained (i.e. lab name), the calibration program and the protocol for quality assurance OR state that no new dates are provided.*

☒ Tick this box to confirm that the raw and calibrated dates are available in the paper or in Supplementary Information.

Ethics oversight	No ethics approval was required as the analysis is made up of published data or synthetic and analysed using free software tools

Note that full information on the approval of the study protocol must also be provided in the manuscript.
